# Data Processing Optimization in Untargeted Metabolomics of Urine Using Voigt Lineshape Model Non-Linear Regression Analysis

**DOI:** 10.3390/metabo11050285

**Published:** 2021-04-29

**Authors:** Kristina E. Haslauer, Philippe Schmitt-Kopplin, Silke S. Heinzmann

**Affiliations:** 1 Research Unit Analytical BioGeoChemistry, Helmholtz Zentrum München, German Research Center for Environmental Health, D-85764 Neuherberg, Germany; kristina.haslauer@tum.de (K.E.H.); schmitt-kopplin@helmholtz-muenchen.de (P.S.-K.); 2 Chair of Analytical Food Chemistry, Technical University Munich, D-85354 Freising-Weihenstephan, Germany; 3 German Center for Diabetes Research, Ingolstädter Landstraße 1, D-85764 Neuherberg, Germany

**Keywords:** NMR, metabolomics, data processing, voigt-fitting

## Abstract

Nuclear magnetic resonance (NMR) spectroscopy is well-established to address questions in large-scale untargeted metabolomics. Although several approaches in data processing and analysis are available, significant issues remain. NMR spectroscopy of urine generates information-rich but complex spectra in which signals often overlap. Furthermore, slight changes in pH and salt concentrations cause peak shifting, which introduces, in combination with baseline irregularities, un-informative noise in statistical analysis. Within this work, a straight-forward data processing tool addresses these problems by applying a non-linear curve fitting model based on Voigt function line shape and integration of the underlying peak areas. This method allows a rapid untargeted analysis of urine metabolomics datasets without relying on time-consuming 2D-spectra based deconvolution or information from spectral libraries. The approach is validated with spiking experiments and tested on a human urine ^1^H dataset compared to conventionally used methods and aims to facilitate metabolomics data analysis.

## 1. Introduction

The field of metabolomics aims to study the complex mixture of metabolites in any tissue or organism and is widely used in several research fields for biomarker discovery, in nutritional studies or to personalized medicine-related scientific questions [1,2,3,4]. Two main spectroscopic methods dominate this field, namely mass-spectrometry (MS) and nuclear magnetic resonance spectroscopy (NMR) [5]. Despite the lower sensitivity, proton-NMR spectroscopy has the advantage of directly producing quantitative measures and additionally offers structural information, as well as high reproducibility [6,7,8]. Nevertheless, drawbacks and challenges exist. Proton signals underlie the sensitivity against minor changes in pH or matrix composition, which results in drifts along the chemical shift axis of some metabolites whereby the extend differs between resonances [9,10,11]. This positional noise adds variation to the dataset and therefore affects subsequent analysis. Several alignment algorithms, e.g., recursive segment wise peak alignment (RSPA) [12], address the problem of peak shifting, but they are not optimal. Furthermore, baseline irregularities occur based on spectral artefacts from electronic distortions, incomplete digital sampling or cumulative underlying signals [13]. Metabolites with similar chemical shifts exhibit peak overlap, which also affects further analysis. As metabolomics often aim to identify biomarkers from datasets, which tend to have high variances in metabolite presence and concentration by nature, additional variance should be kept as low as possible. 

Various tools have been published, which circumvent these drawbacks and facilitate data analyses, utilizing defined metabolite libraries and fitting peaks, according to their pre-defined multiplicities and characteristics within defined matrices [14,15,16]. A comprehensive overview can be found in Bingol et al. (2018) [5]. These methods have been shown to produce reliable and quantitative results, but rely on databases, which are often limited to a specific biofluid, and fail to extract unknown informative features. Non-commercial untargeted approaches are made up from two main strategies, full spectra analysis, which uses all points of the spectrum and various binning methods, where equidistant binning with a binsize of 0.01 − 0.001 ppm is prevalent [17]. Both methods are affected by peak shifting, baseline influence and signal overlap, which adds uninformative noise. Furthermore, full spectra analysis results in large datasets which are bulky to process. Binning has the advantage of a reduction in dimensionality, which speeds up analysis, but limits the ability of detecting metabolites of interest as some peaks may shift between bins through the dataset. In particular, binning either sums up all data points within a certain bin or determines the area under the curve (AUC), significant changes in minor peaks may be covered by general variance caused by baseline differences or signal overlap. To address these issues an easy-to-use and straightforward processing step is introduced, which is based on a peak-picking algorithm followed by a Voigt lineshape model fitting. In theory, NMR peaks are Lorentzian. However, slight variations in peak linewidth (e.g., due to shimming imperfection) lead to random error in the Lorentzian model. To account for this issue, a Voigt lineshape model, which is a convolution of Lorentzian and Gaussian shapes, has shown to be more accurate [18,19]. As both binning and full spectra analysis are widely used methods for NMR metabolomics processing, the performance of the Voigt fitting workflow is validated by comparison to these methods. The introduced workflow aims to provide an enhanced processing method that extracts information from NMR spectra without limitations set by the necessity of pre-defined databases. 

**Figure 1:**
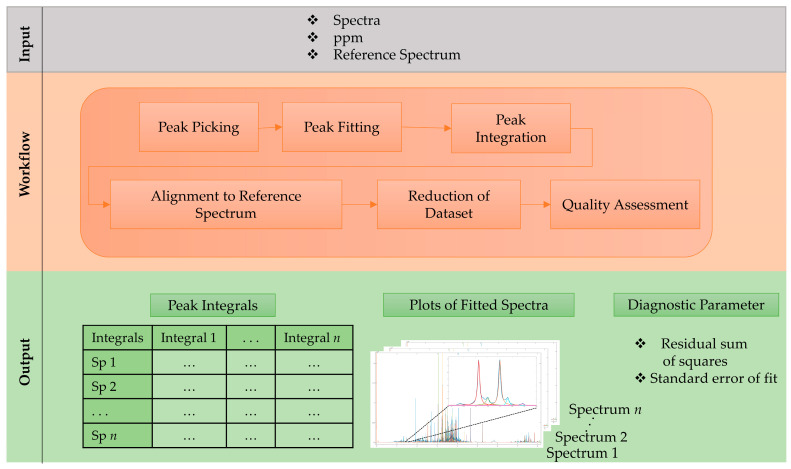
Step-by-step schematic workflow description for non-linear peak fitting based on Voigt line shape model.

**Figure 2:**
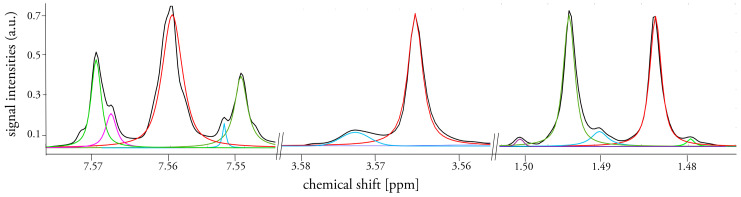
Typical fit results for an exemplary urine spectrum in three regions where signals overlap and/or small peaks are present; initial spectrum is shown as black line, fitted peaks are depicted in colored lines.

To overcome these drawbacks we introduce an untargeted workflow for complex NMR spectra, which consists of 6 main steps that are shown in Figure 1. As with the input information, the workflow uses aligned, normalized NMR spectra and a reference spectrum (e.g., quality control or mean spectrum (mean (x)). First, a peak picking approach is performed on the full dataset for every single spectrum by finding all local maxima. This is followed by an optional noise reduction step, where all peaks with a net intensity between the local maximum and the neighboring minimum are discarded. Therefore, an adjustment for the noise level, especially in regions with a baseline above zero, as well as in overlapping peak regions is achieved. In the next step, the non-linear peak fitting algorithm constructs Voigt line-shaped approximated peaks to the experimental data by optimizing amplitudes, peak maxima, the ratio between Gaussian and Lorentzian and peak width. Peak fitting is based on the lsqcurvefit function inbuilt in MATLAB, employing a trust-region-reflective algorithm. In the following step, the AUC of fitted peaks are calculated over a defined integration range (i.e., multiple of optimized peak width). The chemical shift (i.e., their local maxima) of these peak integrals vary slightly, even in aligned datasets. Therefore, peak shifts are adjusted to the reference spectrum by an alignment step that iterates through every processed spectrum to find peaks within a user defined peak shift window. The generated dataset of integrated peaks can now be further reduced by applying a frequency filter to exclude peaks that are present in less than a set percentage in the dataset. Finally, the workflow gives as output: a list of peak integrals, a plot of each fitted spectrum and quality metrics, such as the residual sum of squares and the standard error of fit for the fitting parameters. These metrics, as well as the graphical results (see Figure 2) allow a quality assessment of the obtained data and consider it for further processing, e.g., applying weighting function. 

## 2. Results

### Error Estimation over Matrices

**Figure 3:**
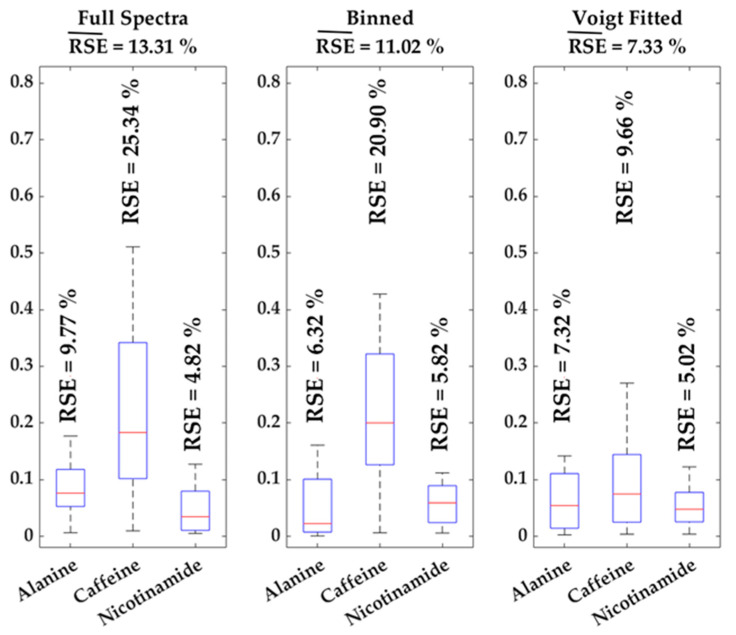
Boxplots of standard errors of relative quantification for all three spiked metabolites and methods, individual relative standard errors (RSE) are given as well as the mean RSE ($\bar{\mathrm{RSE}})$ for each method.

Efficient metabolomics analysis aims to uncover patterns and trends within the data. However, in NMR metabolomics analysis, such trends are often covered by background noise and peak shifts. The comparability of the introduced approach with conventionally used methods, full spectra (i.e., peak height) and binned data analysis (i.e., AUC of spectral bin), is shown using a standard addition of three metabolites (Alanine, Caffeine and Nicotinamide) with three spiked concentrations in four different urine samples, which results in 12 data points. The data are used to calculate a standard curve for every method. These equations were used to re-calculate the concentration for all 12 individual values. Boxplots (see Figure 3) are employed to illustrate the error proneness for all three methods sorted by the respective metabolite including standard errors. Averaging the mean standard errors over the three investigated metabolites for every method gives total mean relative standard errors ($\bar{\mathrm{RSE}})$ (13.31% for full spectra analysis, 11.02% for binned data and 7.33% using Voigt fitted data). The metabolites differ, shown in this study, in their chemical shift, their tendency to shift and/or overlap, thus, the large span of relative errors (see Figure 3) is somewhat expectable. Overall, these results indicate that applying the Voigt fitting algorithm does not artificially increase the variation in comparison to full spectra and binned data analysis. The main advantage of the Voigt function integral lies in the removal of background noise, illustrated by large improvements in RSE for the overlapped signal of caffeine and similar RSE for relatively large and/or non-overlapped peaks, such as alanine and nicotinamide and is therefore applicable for usage in metabolomics approaches.

**Figure 4:**
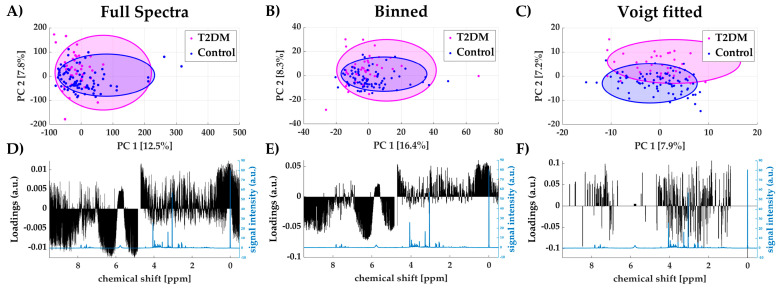
Scores plots of PC1 and PC2 using full spectra (**A**), binned data (**B**) and Voigt fitted data (**C**) including 95%-confidence ellipses for each group (Type 2 diabetes mellitus (T2DM) and control); loadings plot for PC1 for all three methods (black) with reference spectrum (blue) (**D**–**F**).

The publicly available dataset MTBLS1 [20] from the MetaboLights repository [21] was processed using the full spectra, binned data and Voigt fitted data approach. The MTBLS1 study contains 132 spectra of human urine samples from patients with Type 2 diabetes mellitus (T2DM) and a control group. A principal component analysis (PCA) was performed to determine the areas of highest variance using the different data processing methods as input data. In Figure 4A–C scores plots of the first two principal components (PC1 and PC2) are shown for all three methods, which are colored according to their groups (T2DM/Control). Both full spectra and binned data scores plots fail to separate healthy and diseased individuals. Using Voigt fitted peak integrals as the input data for PCA, a separation can be observed between patients with type 2 diabetes mellitus (T2DM) and the control group, which was intuitively expected. The loadings plot of full spectra analysis (Figure 4D) shows that the majority of variance arises from high amplitudes in the upfield region (δ < 1 ppm), around the residual water signal (δ~4.7 ppm) and in the very downfield region (δ > 8.5 ppm). In these regions, generally, few or no peaks occur in urine samples and they are mainly dominated by bare baseline. Similar results are observed for binned spectra, where high variations in uninformative regions also dominate the principal components (Figure 4E). The Voigt peak fitting approach reduces the spectral data to informative peak areas. Here, the loadings for PC2 (Figure 4F) show high variance of urinary glucose levels between patients and the control group (ratio between mean relative intensities (a.u.): 2.09 T2DM/Control, which is expected in an unmedicated cohort. Intriguingly, this obvious information could not be extracted from PCA loadings of the full spectra and binned data analysis, as it was covered by background noise. 

In summary, these results show that using Voigt fitted peak integrals instead of the whole spectrum (as is or binned) allows a crucial reduction of noise, and thus, facilitate the unsupervised data analysis. 

**Figure 5:**
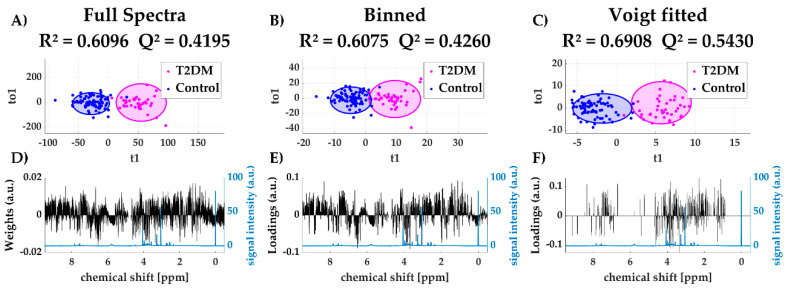
Scores plots of predictive and orthogonal variation using full spectra (**A**), binned data (**B**) and Voigt fitted data (**C**) including 95%-confidence ellipses for each group (Type 2 diabetes mellitus (T2DM) and control); loadings plot for first predictive component for all three methods (black) with reference spectrum (blue) (**D**–**F**).

Supervised methods, such as orthogonal projection on latent structures (OPLS) [22], aims to separate the total variation within a dataset into a predictive (i.e., information related to the sample class) and an orthogonal (i.e., unrelated) component. This method is generally accepted to exclude non-informative noise and thus uncover the relevant information related to the sample class. In Figure 5A–C OPLS discriminant analysis scores plots are shown including their R^2^ and Q^2^ values. Although all three methods yield a valid model to distinguish diabetic and non-diabetic individuals, both R^2^ and Q^2^ are higher using the Voigt fitted dataset. Furthermore, the loadings plots of the predictive component (Figure 5D,E) still show a considerable influence of non-informative regions (~0 ppm, ~5 ppm, >8.5 ppm). 

Overall, these results indicate that the noise reduction achieved by applying the introduced peak fitting using a Voigt approximation enables a more convenient analysis of NMR metabolomics datasets. Through the reduction of the dataset, a yet inevitable visual inspection of results becomes more simple and false positive results caused by baseline differences are reduced. Furthermore, the impact of different data analysts is largely reduced. 

## 3. Discussion

The field of untargeted NMR metabolomics became increasingly important over the past few years. However, effective and reliable data processing remains a bottleneck. The majority of studies published in the field of untargeted metabolomics rely on, either full spectra analysis or on different binning methods. Although NMR is generally highly reproducible, minor changes in baseline intensities may occur due to accumulation of underlying signals, as well as line broadening due to inhomogeneity of the magnetic field. Both conventionally used methods are limited in their ability to compensate for this non-informative variance. Nevertheless, a reduction of this noise is a crucial aspect in uncovering relevant variance and allow identification of biomarkers. The introduced approach aims to improve the efficiency of untargeted NMR metabolomics data analysis by using a peak fitting approach, based on a Voigt line shape model approximation in a least square sense, along with alignment of peak integrals to a reference spectrum. Peak fitting reduces the noise driven bias by reduction of the data. Regions containing mere baseline or very small peaks below a defined S/N ratio are excluded from further analysis, and thus, reduce the influence of the measurement error, which is usually relatively large for small values, and the irrelevant variation within the data. A comparison of all three data processing methods (full spectra, binning and Voigt fitting) demonstrates the reduced extend of noise influence of analysis performed using Voigt fitted data compared to conventionally used processing methods for both unsupervised and supervised analysis methods. A significant influence of noise within the first principal components is a well-known feature and generally accepted as fact within the NMR metabolomics community. An orthogonal PLS is typically the method of choice to segregate this noise from the biological variation of interest. Although an orthogonal filter is applied, a significant influence of non-informative variance is demonstrated using conventional data processing. Several research articles have been published, optimizing both integration and data reduction in various biofluids. From these, several approaches need input data, such as a predefined target list or spectral libraries and deliver a targeted metabolomics output, as reviewed by Bingol et al. [5], while our approach remains untargeted. Other workflows, such as SigMa [23] require extensive compound libraries. Applied to serum and plasma samples, Takis et al. [24] introduced a deconvolution-free integration method, SMolESY, which enables a suppression of the macromolecular background, which is particularly important in blood samples. Its application to urine samples remains unclear, as plasma, unlike urine, does not face extensive peak shifting. Our project contributes to the continuous progress in the field of optimized data processing in untargeted NMR metabolomics.

Voigt-fitting decreases the chance of detecting false positive markers by general data reduction and simplifies the interpretation, and analysis of loadings, respectively weights. Nevertheless, thresholds for S/N and frequency filter must be adjusted carefully to avoid rejection of relevant signals. The Voigt fitting approach was developed and tested on human urine samples as representative biofluid for complex mixtures. However, this method can also be adapted and optimized for other biological matrices. 

The relevance of improved data processing methods is clearly supported by the comparison of performance of data processing methods in this work. Peak fitting using a Voigt line shape model has been demonstrated to enhance the power of statistical analysis in contrast to conventionally used methods. The used script is written in MATLAB R2020a and can be obtained for implementation by contacting the corresponding author. 

## 4. Materials and Methods

### 4.1. Study Cohort

For illustration of performance improvement the fitting approach in comparison with full spectra and binning approach the MTBLS1 dataset (raw spectra) from the MetaboLight repository [21]. The MTBLS1 dataset consists of 48 samples from unmedicated patients with Type 2 diabetes mellitus (T2DM) and 84 samples from healthy individuals as control group. The study was conducted to examine urinary metabolic changes in patients with T2DM in comparison to the control group. Details about sampling, sample preparation, acquisition along with main findings are available in the original manuscript [20]. 

### 4.2. Validation Dataset

The error estimation of the three tested methods was calculated using four different urine samples each spiked with l-alanine, Caffeine and Nicotinamide in three concentrations by comparing the results to peak height in full spectra analysis and AUC in binned spectra analysis. l-alanine was used because its resonance appears in a non-crowded region and shows a distinct doublet as easy-to-integrate standard. Caffeine has resonances in a crowded region where baseline effects do occur (3–4 ppm) and Nicotinamide causes resonances in the downfield area to comprehensively cover the whole spectrum. A stock solution of 1 mg mL^−1^ H_2_O was prepared. A total of 135 µL urine was combined with either 5, 10 or 15 µL stock solution resulting in an addition of 5, 10 and 15 µg standard. The samples were then filled up to a total volume of 150 µL, 50 µL 1.5 M K_2_PO_4_-buffer (pH 7.4) containing 0.1% Trimethylsilylpropionic acid (TSP) in 100% D_2_O was added, samples were thoroughly vortexed and centrifuged at 4 °C for 10 min at 12,700× *g*. A volume of 180 µL of supernatant was transferred into 3-mm NMR tubes. Samples were measured immediately after preparation. 

### 4.3. NMR Data Acquisition and Processing

The samples were analyzed on a Bruker 800 MHz spectrometer operating at 800.35 MHz equipped with a quadrupole inverse cryogenic (QCI) probe probe (Bruker BioSpin, Rheinstetten, Germany). A total of 256 scans were recorded into 64 K datapoints with a spectral with of 16 ppm and a 90° pulse of 13 µs. All spectra were acquired at 300 K using a standard 1D-pulse sequence with water suppression (noesygppr1d) during an recycle delay of 4 s, an acquisition time of 3 s, and a mixing time (tm) of 200 ms. Spectra were manually phased and baseline corrected in TopSpin 3.6.1 (Bruker BioSpin, Rheinstetten, Germany). 

### 4.4. Data Processing

Spectra were imported into Matlab software (R2020a; Mathworks) for data processing with a resolution of 2.5 × 10^−4^ ppm, resulting in 44,001 data points per spectrum (−1 to 10 ppm). The water region was removed (δ 4.70−4.85 ppm). Spectra were aligned using a recursive segment-wise peak alignment (RSPA) algorithm [12], probabilistic quotient normalization was used to account for biological variation in urine dilution [25]. To compare the performance of the here introduced approach, two conventionally used processing methods (full spectra analysis and binning of spectra) were used as state of the art reference for untargeted metabolomics [4,26]. For full spectra analysis the data matrix was used as is after water removal and alignment resulting in a 132 × 43,400 matrix. Binning was performed by dividing every spectrum in equidistant buckets with a bin width of 0.01 ppm and determining the area under the curve (AUC) for every bin by trapezoidal integration. The resulting data matrix has a size of 132 × 1085. Peak fitting was performed using the above described workflow and is resulting in a 132 × 432 data matrix. A threshold was set to a minimum of 30% abundance through the samples with a signal to noise (S/N) ratio above 5. 

Principal component analysis (PCA) was performed in Matlab software (R2020a; Mathworks) using unit variance (UV) scaling prior to analysis. 

Orthogonal projection on latent structures (OPLS) discriminant analysis was performed according to the method described in Cloarec et al. (2005) [27].

## Data Availability

Publicly available datasets were analyzed in this study. This data can be found here: https://www.ebi.ac.uk/metabolights/MTBLS1/descriptors.
